# The sleep as a predictor of musculoskeletal injuries in adolescent athletes

**DOI:** 10.5935/1984-0063.20220055

**Published:** 2022

**Authors:** Fernanda Viegas, Juliana Melo Ocarino, Luísa de Sousa Freitas, Marina Costa Pinto, Lucas Alves Facundo, Adriana Souza Amaral, Samuel Silva, Marco Túlio de Mello, Andressa Silva

**Affiliations:** 1 Universidade Federal de Minas Gerais, Sports Department - Belo Horizonte - Minas Gerais - Brazil; 2 Universidade Federal de Minas Gerais, Physiotherapy Department - Belo Horizonte - Minas Gerais - Brazil

**Keywords:** Sleep, Athletic Injuries, Sports, Athletes, Adolescent

## Abstract

**Objectives:**

Sleep is essential for musculoskeletal and cognitive recovery. Adolescent athletes tend to sleep poorly compared to adults and it may predispose them to sports injuries. Our aims are to estimate whether the quantity/quality of sleep are associated with sports injuries in adolescent athletes and to compare the quantity/quality of sleep between the training and competition seasons, and the school vacation period.

**Material and Methods:**

It was a cohort study with 19 track and field athletes of both sexes, aged between 12 and 21 years. We evaluated their sleep-wake habit through actigraphy during three phases: 1 - mid-season, 2 - competition, and 3 - school vacation. The previous six months injury history and the occurrence of injuries in a six-month follow-up were recorded. Logistic regression and variance analysis were performed. The significance level used was 0.05.

**Results:**

Wake after sleep onset (WASO) predicted previous injuries (OR=1.144) and time awake (TA) predicted injury occurrence (OR=0.974). TA decreased from phase 2 to phase 3 (*p*=0.004), total sleep time (TST) increased from phase 2 to phase 3 (*p*=0.012), and WASO decreased between phases 1 and 2 (*p*=0.001) and between phases 1 and 3 (*p*=0.025).

**Conclusion:**

Our study demonstrated that the quantity and quality of sleep were associated with musculoskeletal injuries in adolescent track and field athletes. Previous injuries were predicted by WASO and the occurrence of injuries was predicted by TA. Furthermore, during the vacation period they had lower TA and WASO, and higher TST than on school days.

## INTRODUCTION

Sleep is an active process considered a functional, cyclical, and reversible state, which is important for the maintenance of physiological and cognitive functions of human beings^1^. Good sleep quality predicts good mental and physical health^2^ and it is recognized as one of the most effective recovery strategies^3^. In recent years, the general population has experienced a reduction in sleep duration which can generate potential risks to the individual’s health^4^. In this sense, the interest about the athletes’ sleep has increased, considering its beneficial effects to musculoskeletal recovery^3^.

Athletes have a greater need for sleep compared with the general population; however, sleep restriction is common in athletes especially before competitions and it can significantly impact their performance^1^. Some factors may explain the worsening in sleep quality in athletes such as increased cortisol concentrations^5^, increased sympathetic activity^6^, increased central body temperature^7^, presence of muscle pain^8^, as well as anxiety and thoughts about sports competition^9^. Thus, typical changes in hormone secretion patterns induced by sleep debt can decrease protein synthesis and increase protein degradation, impairing skeletal muscle integrity^4^,^10^; therefore, sleep deprivation can affect the process of sports recovery in this population^11^.

Adequate sleep duration, good sleep quality, regularity of the sleep-wake cycle, and absence of sleep disorders are all factors that contribute to achieving healthy sleep in adolescents^12^. Several benefits of healthy sleep in adolescents include general health, cardiovascular, metabolic, mental and immunological aspects, and to their body development as well^2^. A consensus of the American Academy of Sleep Medicine states that adolescents should sleep between 8 and 10 hours per night^13^, however, considering that they are in a crucial period for brain development^14^, some authors argue that adolescents should sleep at least 9 hours a night^15^. On the other hand, when adolescents are under a state of reduced sleep duration, impairments in motor and cognitive function may occurr^16^. Despite recommendations, insufficient sleep is highly prevalent among adolescent athletes^17^ and could be explained by habits such as staying awake later and waking earlier in the morning due to school commitments^18^. Due to biological and environmental factors, is expected a gradual delay in chronotype during adolescence, reaching its peak at the end of puberty, which means a preference for going to bed later and waking up later^19^. Therefore, the school start time in many countries including Brazil, contradicts this factor and it requires adolescents to wake up in the early morning, inducing a situation of chronic sleep restriction^20^. Added to the sleep changes expected by age, the need of managing the sports demands with school and/or even with work demands may predispose adolescent athletes to an even poorer sleep condition^21^. In this sense, a recent study by Patel et al. (2020)^22^ found through actigraphy measurements a mean total sleep time (TST) on weekdays of 6.04 hours in 17 adolescent athletes of track and field from the USA. However, the mean TST on weekends was 7.01 hours. Furthermore, the authors found a positive correlation between TST and performance on neurocognitive tasks.

As quantity and quality of sleep might influence muscle recovery^4^,^9^ insufficient sleep may increase the occurrence of musculoskeletal injuries in athletes^1^. Milewski et al. (2014)^17^ using subjective measures found that adolescent athletes who slept less than 8 hours per night had a 1.7 increase in the risk of being injured. Moreover, a recent study conducted with elite soccer players showed that athletes with worse sleep quality presented a greater amount and higher severity of musculoskeletal injuries^23^.

Although some studies have investigated the associations between sleep and sports injuries, most of them used subjective methods such as questionnaires to evaluate sleep. Thus, the literature lacks information on whether some specific sleep variables measured through objective instruments could predict musculoskeletal injuries in adolescent athletes. In addition, it is important to understand the sleep characteristics of adolescent athletes and their patterns on school days and vacation periods since it could have some implications for their training programs. The aim of the present study is to estimate whether the quantity and quality of sleep assessed by objective measures would be associated with musculoskeletal injuries in adolescent athletes. Our secondary aim is to compare the quantity and quality of sleep between the training and competition seasons, and the school vacation period.

## MATERIAL AND METHODS

The study was approved by the research ethics committee of the Federal University of Minas Gerais on number 64492016.8.0000.5149 and all athletes signed the free and informed consent form.

### Participants

The sample was obtained by convenience. Initially, we recruited 30 athletes of both sexes (19 males and 11 females) aged between 12 and 21 years, however, 11 athletes were excluded from the final sample because they did not participate in all procedures and/or did not use the wrist actigraph for more than seven consecutive days. Thus, the final sample was composed of 19 athletes (13 males and 6 females). They were recruited from the track and field teams (categories - 100, 200, and 400 meters) of the Sports Training Center of the Federal University of Minas Gerais and they participated in competitions at local, regional and/or national level in the last 6 months.

### Procedures and evaluation

#### Sleep

The sleep evaluations were performed in three phases:

Phase 1 (August 2018) - mid-season of sports season.

Phase 2 (October 2018) - competitive period.

Phase 3 (January 2019) - school vacation period maintaining sports training.

Sleep analysis was performed using an *Actiwatch* 2 wrist activity monitor actigraph (*Philips Respironics*^®^, Andover, MA), an instrument that allows continuous monitoring of rest-activity cycle in different populations^24^. In athletic populations, actigraphy has been considered the preferred method to measure sleep objectively as it has minimal impact on sleep and training routines^25^. The data collected by the wrist actigraph were stored in its internal memory, then they were transferred to a computer and analyzed through the software Action-W version 02, Ambulatory Monitoring Inc^®^, in which a graphic record was generated in the actogram of each athlete. The variables analyzed by the device were time awake (TA), TST, sleep latency (LAT), sleep efficiency (SE), and awakenings after sleep onset (WASO). LAT is defined as the transition period between wakefulness and sleep onset, and for all age groups the recommended value for LAT is ≤15 minutes, which indicates a good sleep quality^2^. SE is established as the percentage of the proportion between TST and the time that the individual remains in bed, and the current recommendation for good quality sleep is SE ≥ 85%^2^. WASO reflects sleep fragmentation, and the literature recommends WASO values ≤20 minutes to be considered good sleep quality^2^.

The athletes were instructed to wear the actigraph on the non-dominant wrist and to use it continuously for 10 days maintaining their usual sleep-wake pattern during the study period. In addition, the athletes were asked to fill out a sleep diary to record the sleep-wake cycle and to check when sleep episodes and nap periods started and ended, as well as to record the moments when the wrist actigraph was removed. The procedures were the same in all three evaluation phases.

#### Musculoskeletal injuries

To characterize the athletes’ previous injuries, we used a modified version of the *Fédération Internationale de Football Association* (FIFA) questionnaire^26^ to collect the history of injury considering the six months prior to the first sleep assessment. The questionnaire modifications were made along with the technical committee: 1) the question related to the practice of football was removed: “Was it caused by contact or collision?”: “with another player”; “yes, with the ball”; “yes, with another object (specify)”; 2) three new questions were added. A question regarding the practice of track and field: “Was the injury caused by a fall or contact?” with the following answers: “no”; “yes, by fall”; “yes, contact with the barrier”; “yes, contact with another athlete”; “yes, contact with another object (specify). In addition to a question about the withdrawal from sports activities: “Did you need to interrupt sports activities?” with the following answers: “total absence”; “partial restriction”; “physiotherapeutic treatment without withdrawal from sport activities”. Finally, a question regarding the physical therapy assessment was also added: “Was the injury evaluated by a physical therapist?” with the following answers: “no”; “yes”.

The questionnaire characterizes each sports injury in relation to the date of the event and the return to sport, part of the body, injury type, medical diagnosis, recurrence, mechanism, and causes^26^. According to Fuller et al. (2006)^26^, injury is conceptualized as any physical complaint suffered by an athlete that results from a training or a competition, regardless of the need for medical attention and/or withdrawal from sports activities.

To record and monitor the occurrence of injuries, an electronic form was used in which the athlete’s name, gender, age, sport category and dominant limb were recorded. Regarding injuries data, the classification, location, type, diagnosis, recurrence, mechanism, time away from sports practice^26^, and duration of physiotherapy treatment were recorded.

#### Statistical analysis

The descriptive analysis of the variables was presented quantitatively, as well as the means and standard deviation were calculated according to the data obtained. The variables related to musculoskeletal injuries (previous injuries and occurrence of injury) were settled as dichotomous variables (1 = yes; 2 = no). The Shapiro-Wilk test was applied to verify the normality of the data. With the purpose of estimating the prediction of musculoskeletal injuries, it was performed a binary logistic regression having as independent variables the variables related to sleep such as TA, LAT, TST, SE, and WASO, after observing the absence of multicollinearity by the value of r among the variables. Finally, to compare the means of sleep variables, in the three evaluation phases, variance analysis (ANOVA) of repeated measures was used. When necessary, Bonferroni’s post hoc was used. The significance level used was 0.05. The analyses were performed in the software SPSS (version 20.0).

## RESULTS

The characteristics of the sample are shown in Table 1. Thirteen boys and six girls composed the final sample. In relation to the school shift, 12 of them studied in the morning (07:30 to 12:00) and 7 studied at night (19:00 to 22:30).

**Table 1:** Sample characteristics (n=19).

Age (years)	Body weight (kg)	Height (m)	BMI (kg/m^2^)	Weekly training frequency	Sex	School shift
16.89 ± 2.75	62.35 ± 8.33	1.72 ± 0.11	21.1 ± 1.42	4.63 ± 0.77	13 male athletes	12 morning shift
					6 female athletes	7 night shift

Notes: Values represented in mean and SD (±). Abbreviations: BMI = Body mass index.

Binary logistic regression was performed, using sleep variables as independent variables, such as TA, LAT, TTS, EF, and WASO. In this model, the dependent variable was previous injuries and the occurrence of musculoskeletal injuries, in a dichotomous way (1 = yes; 2 = no). This process was repeated with the variables of phases 1, 2 and 3. According to the logistic regression, the model containing WASO was statistically significant [X^2^(1)=9.023; *p*=0.003; R^2^_Negelkerke_=0.517] with 84.2% of correct classification. WASO was a significant predictor of previous injuries (OR=1.144) in phase 1. The model containing the selected sleep-related variables (TA, TST, and LAT) was statistically significant [X^2^(1)=6.472; *p*=0.011; R^2^_Negelkerke_=0.422] with 78.9% of correct classification. TA was a significant predictor of the occurrence of musculoskeletal injuries (OR=0.974) in phase 2.

Table 2 shows the descriptive results of the variables related to musculoskeletal injuries in the group, such as the number of injuries and physiotherapy sessions in all evaluation phases (August 2018, October 2018, and January 2019).

**Table 2:** Descriptive data for the variables related to musculoskeletal injuries: number of injuries and number of physiotherapy sessions in phases 1, 2 and 3 (n=19).

	Phase 1	Phase 2	Phase 3
**Number of injuries**	9	6	4
**Number of physiotherapy sessions**	23	23	5

Notes: Phase 1 = August 2018 (mid-season of the sports season); Phase 2 = October 2018 (competitive period); Phase 3 = January 2019 (end of sports season and school vacation period).

After analyzing the injury form, variables such as the need to be withdrawn from sports practice, the region and side of the body that was injured, the mechanism of injury and whether the injury had been caused by overuse or trauma were collected during the three-month training period and the vacation period (Table 3).

**Table 3:** Characteristics of retrospective and prospective injuries: withdrawal from sport practice, body part, body side, injury mechanism and overuse/trauma (n=19).

Characteristic of injuries	Relative frequency (%)
Withdrawal from sport practice	Partial (7.4%)
	Physiotherapeutic treatment without withdrawal from sports activities (92.6%)
Body region	Neck/cervical (3.7%)
	Lumbar/sacrum/pelvis (14.8%)
	Shoulder/clavicle (7.4%)
	Wrist (3.7%)
	Hip/groin (3.7%)
	Thigh (29.6%)
	Knee (3.7%)
	Leg/Achilles tendon (25.9%)
	Ankle (7.4%)
Body side	Dominant (44.4%)
	Non-dominant (7.4%)
	Bilateral (25.9%)
	Not applicable (22.2%)
Injury mechanism	Fracture (3.7%)
	Sprain/ligament injury (3.7%)
	Stretch/tension/injury/muscle cramp (88.9%)
	Tendon injury/rupture or tendinosis (3.7%)
Overuse/trauma	Overuse (92.6%)
	Trauma (7.4%)

Notes: Values represented in relative frequency (%).

Table 4 describes the sleep variables in the three evaluation phases. For TA, the results revealed an significant effect and it was shown that the TA decreased significantly from phase 2 to phase 3 [F_(2.36)_=6.512; *p*=0.004]. For LAT, no statistically significant differences were found between the phases [F_(2.36)_=1.678; *p*=0.201]. For the TST, the results revealed a time effect, so we can affirm that the TST increased significantly from phase 2 to phase 3 [F_(2.36)_=5.062; *p*=0.012]. For SE, no statistically significant differences were found between the phases [F_(2.36)_=0.824; *p*=0.447]. For WASO, the results revealed a time effect. Furthermore, we observed that the WASO decreased significantly between phases 1 and 2 [F_(2.36)_=14.531; *p*=0.001], and between phases 1 and 3 [F_(2.36)_=14.531; *p*=0.025].

**Table 4:** Descriptive data for sleep variables - awake time, sleep latency, total sleep time, sleep efficiency, and wake after sleep onset during ten days of actigraphy monitoring (n=19).

Sleep variables	Phase 1	Phase 2	Phase 3	*p*
**Time awake (min)**	957.29 ± 69.33	980.72 ± 68.48	924.1 ± 63.62^*^	0.004
**Sleep latency (min)**	20.81 ± 10.48	24.16 ± 13.51	18.4 ± 13	0.201
**Total sleep time (min)**	433.01 ± 44.48	416.41 ± 46.44	453.1 ± 56.96^*^	0.012
**Sleep efficiency (%)**	82.5 ± 3.69	83.29 ± 6.53	84.25 ± 4.91	0.447
**Wake after sleep onset (min)**	46.03 ± 12.42	29.21 ± 9.95#	36.14 ± 15.28#	0.001

Notes: Values represented in mean and SD (±) and relative frequency (%). Phase 1 = August 2018 (mid-season of sports season); Phase 2 = October 2018 (competitive period); Phase 3 = January 2019 (end of sports season and school vacation period);

* = Differs from phase 2; #Differs from phase 1.

## DISCUSSION

The aims of this study were to investigate the association between the quantity and quality of sleep measured objectively through actigraphy and musculoskeletal injuries in adolescent athletes, and to compare the quantity and quality of sleep between the training and competition seasons, and the school vacation period. The results showed that WASO was negatively associated and was able to predict the history of previous injuries. An increase in TST and reduction of WASO in the vacation period were observed, concomitant with the reduction of musculoskeletal injuries in this period. In addition, the athletes presented TST, LAT, SE, and WASO values compatible with poor quality sleep in all evaluation phases^2^.

After performing the logistic regression model, we found that WASO was a significant predictor of previous musculoskeletal injuries, as well as TA was a significant predictor of the occurrence of musculoskeletal injuries. Corroborating our findings, Silva et al. (2019)^23^ found that 30% of the musculoskeletal injuries were explained by WASO in a sample of elite soccer athletes. Previous studies that used questionnaire to evaluated sleep found through the logistic regression model that the adolescent athletes who slept the recommended amount reduced the occurrence of musculoskeletal injuries by 61%^27^. Finally, it was observed that by increasing one minute of TA, the athlete was 2.6 times less likely to suffer a prospective musculoskeletal injury. As sports injury has a multifactorial nature^28^ the results of the present study reveal that sleep can be one of the factors related to injury occurrence and should be considered in adolescent athletes.

WASO represent sleep fragmentation. Our findings raise concerns about the presence of complaints of several episodes of awakenings during the night since they may be associated with musculoskeletal injuries in adolescent athletes. Thus, this is an important sleep variable to be taken into consideration in sports practice by the staff professionals, whether in adult athletes based on previous studies^24^ or also in adolescent athletes based on the findings of this study.

When comparing the sleep variables between the evaluation phases, we detected a significant reduction in TA and WASO, and a significant increase in TST during the school vacation period. Also, we found a lower number of musculoskeletal injuries and physiotherapy sessions in that phase. In the sports context, athletes are expected to have episodes of sleep restriction especially due to the frequency, intensity, and volume of training^29^, in addition to pre-competitive anxiety^9^, possibly as occurred in the phase 2 of the present study when they were in a competitive period. Furthermore, when dealing with adolescent athletes, this becomes even more worrisome due to a susceptibility to sleep alterations in this life period^22^,^30^, considering that they must manage sports and school demands^21^.

LAT, SE, and WASO are variables related to sleep quality^2^ and they were measured in this study. The sleep quality of adolescent athletes can be influenced by the combination of school and sports commitments^14^ as well as by their chronotype, depending on the schedules imposed by society (e.g., training shift and school start time)^31^. For example, adolescents presenting an eveningness chronotype might have difficulties in waking up too early to study^32^. At this stage of life, adolescents undergo several biopsychosocial changes, including the sleep-wake cycle, and it is a phase of constant development, in which sleep plays a crucial role^33^. Our sample presented LAT, SE, and WASO values that characterize their sleep as poor quality^2^. LAT is defined as the transition period between wakefulness and sleep onset expressed in minutes, and values ≤15 minutes indicates good sleep quality. In phase 1, a mean LAT of 20.81 minutes was observed; in phase 2, 24.16 minutes; and in phase 3 (vacation), 18.4 minutes. In a study conducted with Brazilian soccer athletes the authors found a mean LAT of 29.65 minutes^23^. However, according to George and Davis (2013)^34^, during adolescence there is an increase in LAT due to hormonal changes. In relation to SE, values ≥85% are considered normal, in phase 1, mean SE of 82.5% was observed; in phase 2, 83.29%; and phase 3 (vacation), 84.25%. In recent studies, Brazilian elite soccer athletes had a mean SE of 81.6%^23^ and young American football athletes showed a SE of 89.85%^35^. Regarding WASO, the literature recommends WASO values ≤20 minutes to have a good sleep quality^2^. The athletes of the present study presented in phase 1 a mean WASO of 46.03 minutes; phase 2, 29.21 minutes; and in phase 3 (vacation), 36.14 minutes.

In addition, we found in phase 1 (training period) a mean TST of 433.1 minutes, which is equivalent to 07h:13min; in phase 2 (competitive period), 416.41 minutes, which is equivalent to 06h:56min; and in phase 3 (school vacation/training period), 453.1 minutes, which is equivalent to 07h:33min. In all phases the mean TST was below the 8 to 10 hours recommended by The American Academy of Sleep Medicine^12^ and it was even worse on school days. Due to the circadian alterations that occur in this period of life, a reduction in TST is often seen in this population^22^,^36^. Particularly in adolescence, they have a preference for later bedtimes and to wake up late as well which associated with the early morning school commitments, it can impair their sleep^36^,^37^. However, the chronotype can also influence the sleep of adolescent athletes and those who present an eveningness chronotype have a delayed sleep due to biological and environmental factors^38^, which was not evaluated by the present study and may have influenced our findings.

According to McKnight-Eily et al. (2011)^39^, the vast majority of adolescents report insufficient sleep durations. In the population of Brazilian adolescents, Bernardo et al. (2009)^33^ identified through subjective methods that about 39% of adolescents had shorter sleep duration. More recent data obtained by questionnaires showed that about 53% of Brazilian adolescents tend to report a short sleep duration with an average of less than 8 hours of sleep per night^40^. In the present study with an adolescent athletic sample, we found a sleep duration of around 7 hours per night measured through an objective instrument, which is consistent with those findings. In addition, as showed by Fullagar et al. (2015)^41^, athletes from individual sports such as track and field tend to report poorer sleep compared to team sports athletes, especially close to competitions. The increase in TST found during the vacation period may be due to the fact that there was no need to manage school and sports commitments in that phase. According to Roberts et al. (2009)^42^, environmental factors such as school demands tend to significantly restrict the duration of sleep in adolescents. Yet, the increase in sleep amount in adolescents during school vacation has already been observed in other studies in a Brazilian population through subjective measures^43^. Regarding LAT and SE, there were no significant differences between the evaluation phases in adolescent athletes in our study, however, school is not the only factor that could influence sleep and other sociocultural aspects can have an impact on it^40^.

Considering the results obtained in our study, adolescent athletes and members of the sports staff should be aware of the quantity and quality of sleep in this population and consider the increased injury risk when athletes are in a condition of sleep restriction and/or low quality of sleep in their training programs.

A possible limitation of the present study was only 6 months of follow-up; however, even in this short period it was possible to record the occurrence of injuries. Moreover, injuries that occurred outside the sports context may not have been captured in the monitoring. Another possible limitation was the quantification of sleep since naps were not taken into account as a source of additional sleep, and only night sleep was considered. Finally, the athletes’ chronotype (morningness, indifferent, or eveningness) was not evaluated and it could influence their sleep quantity.

## CONCLUSION

The quantity and quality of sleep is associated to the risk of musculoskeletal injury in adolescent track and field athletes. In addition, during the vacation period, adolescent athletes had lower time awake, higher TST, and lower sleep fragmentation. These findings emphasize the importance of sleep assessment in the sports context and the impact sleep can have on health and, consequently, on the performance of adolescent athletes. Thus, sleep variables related to sleep quality should be considered by professionals who deal with adolescent athletes, since they may present associations with musculoskeletal injuries and might be worse during school days.
